# Draft Genome Sequence of a Multidrug-Resistant *Acinetobacter baumannii* Strain from Chile

**DOI:** 10.1128/genomeA.00687-15

**Published:** 2015-07-02

**Authors:** Andrés Opazo, Bruno S. Lopes, Patricia García, Mariana Domínguez Yévenes, Celia Lima, Helia Bello-Toledo, Gerardo González-Rocha, Sebastian G. B. Amyes

**Affiliations:** a Medical Microbiology, The Chancellor’s Building, University of Edinburgh, Edinburgh, United Kingdom; b Laboratorio de Investigación en Agentes Antibacterianos, Facultad de Ciencias Biológicas, Departamento de Microbiología, Universidad de Concepción, Concepción, Chile; c Department of Microbiology, School of Medicine and Dentistry, University of Aberdeen, Aberdeen, United Kingdom; d Laboratorio de Microbiología, Departamento de Laboratorios Clínicos, Escuela de Medicina, Pontificia Universidad Católica de Chile, Santiago, Chile

## Abstract

*Acinetobacter baumannii* strain Ab5 was isolated in the year 2007 in Chile, being one of the first multidrug-resistant (MDR) cases reported in the country. Here, we present the very first draft genome sequence of an MDR Chilean strain, which shows the presence of diverse resistance and acquired virulence genes.

## GENOME ANNOUNCEMENT

*Acinetobacter baumannii* is a nosocomial pathogen normally classified as multidrug resistant (MDR). Alarmingly, the number of MDR *A. baumannii* isolates has increased in the last few years, making it a difficult microorganism to treat (1, 2), as infections caused by it are associated with high rates of mortality and long periods of hospitalization (3). The rates of *Acinetobacter* species MDR isolates in Latin America have increased lately, as resistance to imipenem in Chile increased from 0.0% during 1997 to 1999, to 47.4% in the 2008 to 2010 period (4), and 65.8% in 2014 (Antibiotic Resistant Group from Chilean Society for Infectious Diseases, personal communication).

In this work, we studied the genetic features of the *A. baumannii* MDR strain Ab5 through whole-genome sequencing (WGS), as this was one of the first sentinel cases of MDR isolates in Chile. To date, there are no whole-genome sequences of *A. baumannii* strains from Chile available in GenBank. The Ab5 strain was isolated at the Hospital of the Catholic University of Santiago in 2007 from a patient who developed a wound infection. Diverse antibiotics were tested by the disk diffusion method and by the agar dilution test, according to the Clinical and Standards Institute (CLSI) guidelines (5). Ab5 was found to be resistant to ceftazidime, imipenem, meropenem, gentamicin, cefotaxime, ciprofloxacin, cefepime, and piperacillin-tazobactam. The MICs to carbapenems were 8 µg/ml to meropenem and 16 µg/ml to imipenem.

WGS was performed utilizing the Illumina HiSeq 2000 platform. The data generated were assembled using the Velvet assembly pipeline (6), with contigs >200 bp, resulting in 107 contigs, a total length of 3,937,075 bp, and an *N*_50_ contig size of 173,003 bp; meanwhile, the average G+C content was 38.9%, coinciding with the data associated with the *Acinetobacter* genus.

Genome annotation was performed utilizing the Rapid Annotations using Subsystems Technology (RAST) (7), and the IGS Annotation Engine was used for structural and functional annotation (8). Manatee was used to view annotations (http://manatee.sourceforge.net/), while ResFinder (9), PathogenFinder (10), and multilocus sequence typing (MLST) of total genome-sequenced bacteria (11) were utilized for complementary analyzes.

We identified 3,657 coding sequences (CDSs), 57 pseudogenes, 7 rRNAs, and 57 tRNAs. Additionally, Ab5 belonged to sequence type 162 (ST-162), which was previously detected in Brazil. We identified the *bla*_OXA-58_, *bla*_OXA-51_, *aph*(3′)-Via, and the DNA topoisomerase type II genes that might explain its MDR phenotype. Interestingly, we found genes associated with iron acquisition, such as the *pitADC*, *piaABC*, and *piuABC* operons, which have been identified in *Streptococcus pneumoniae*. We also identified 25 pathogenesis-associated operons derived from *Mycobacterium* spp., whereas one detected operon, involved in internalin-like proteins synthesis, may have been imported from *Listeria*.

This first approach reflects the ability of *A. baumannii* to capture a wide range of genes derived from different species, making it a troublesome pathogen to treat in the nosocomial environment.

### Nucleotide sequence accession numbers.

This whole-genome shotgun project has been deposited at DDBJ/EMBL/GenBank under the accession no. LANH00000000. The version described in this paper is version LANH01000000.
